# Super High-Concentration Si and N Doping of CVD Diamond Film by Thermal Decomposition of Silicon Nitride Substrate

**DOI:** 10.3390/ma16175849

**Published:** 2023-08-26

**Authors:** Yong Yang, Yongnian Wang, Huaxin Yan, Chenyi Cao, Naichao Chen

**Affiliations:** 1 State Grid Gansu Electric Power Company Institution of Electric Science and Technology, Lanzhou 730000, China; 2 School Energy and Mechanical Engineering, Shanghai University of Electric Power, Shanghai 200090, Chinagpywyyx@163.com (C.C.); 3 Shanghai Key Laboratory of Materials Protection and Advanced Materials in Electric Power, Shanghai 200090, China; 4 Shanghai Non-Carbon Energy Conversion and Utilization Institute, Shanghai 200240, China

**Keywords:** diamond film, silicon nitride, CVD, graphene oxide, nitrogen

## Abstract

The high-concentration N doping of diamond film is still a challenge since nitrogen is limited during diamond growth. In this work, a novel method combined with the thermal decomposition of silicon nitride was proposed to form the activated N and Si components in the reactor gas that surrounded the substrate, with which the high-concentration N and Si doping of diamond film was performed. Meanwhile, graphene oxide (GO) particles were also employed as an adsorbent to further increase the concentration of the N element in diamond film by capturing the more decomposed N components. All the as-deposited diamond films were characterized by scanning electron microscopy, energy dispersive spectroscopy, Raman spectroscopy, and X-ray photoelectron spectroscopy. For the pure diamond film with a growth time of 0.5 h, the N and Si concentrations were 20.78 and 41.21 at%, respectively. For the GO-diamond film, they reached 47.47 and 21.66 at%, which set a new record for super high-concentration N doping of diamond film. Hence, thermal decomposition for the substrate can be regarded as a potential and alternative method to deposit the chemical vapor deposition (CVD) diamond film with high-concentration N, which be favorable for the widespread application of diamond in the electric field.

## 1. Introduction

Diamond is increasingly used in electronic devices, such as new semiconductors, high-power transistors, quantum and sensor fields [1,2], and high-speed switches [3,4] due to its low dielectric coefficient, large band gap, high thermal conductivity, high electron mobility, and high breakdown field, with which the energy loss can be considerably reduced [5]. Notably, pure diamond does not have polarity; thus, doping some elements is a promising option to polarize it [6]. At present, the technology of doping boron into diamond films is relatively mature for purpose of preparing p-type semiconductors, while n-type diamond semiconductors are still being explored [5,7]. The main donor elements of n-type diamond semiconductors are Li, Na, N, S, P, and O elements, among others. Among them, most studies have focused on the phosphorus (P)-doped and nitrogen (N)-doped n-type diamond semiconductors [8,9]. In the past decades, the P-doped n-type diamond semiconductors have attracted the most scholarly and engineering interest, which is due to the fact that the lower activation energy of the P donor element (*E*_d_(P) = 0.57 eV), as compared with the N donor element (*E*_d_ (N) = 1.7 eV) [10], is beneficial for the cost and practicality of deposition applications. However, the large difference in the covalent radiuses between the P atom (*r*_P_ = 1.06 Å) and the C atom (*r*_C_ = 0.77 Å) will lead to defects and the deformation of crystalline grains, causing terminal failure in n-type diamond semiconductors. On the other hand, the covalent radius of the N atom (*r*_N_ = 0.74 Å) is very close to that of the C atom [11,12]. Hence, N doping of chemical vapor deposition (CVD) diamond coatings is essential to develop an alternative and promising method to improve n-type diamond semiconductors.

N-doped diamond coatings have mostly been synthesized by the traditional CVD technique combined with nitrogen-containing chemical reactions. Many studies [13,14] have suggested that Si_3_N_4_ can be decomposed thermally at the annealing temperature of 650–820 °C to diffuse the elemental N and Si inside the metal in the metal–substrate interaction configurations. The experimental results show that N-doped diamond has great potential for visible-light photodetector applications in harsh environments [15]. The lightly nitrogen-doped (N-doped) layer can act as an n-type semiconductor, which is effective in diamond Schottky-pn diodes (SPNDs), similar to a phosphorus-doped n-type layer [16]. The stabilization time of the high-power diamond optoelectronic switches with even a small amount of N doping was much faster than that without N doping [17]. Hence, more efforts were put into the high-concentration N doping of diamond film to explore the unknown semiconduction properties [18]. It has been reported that the bulk resistivity of diamond films synthesized with 1.0% nitrogen doping is lower than in the case of 0.5% [19]. The electrical conductivity can be enhanced for an increase in the nitrogen content [20], and the diamond film with a nitrogen content of 7.9 at.% possessed an n-type condition with an electrical conductivity of 18 S/cm at 300 K [21]. Hence, the high-concentration N doping of diamond film will become an important semiconductor in the near future [22].

Chemical vapor deposition (CVD) is considered to be a well-established and convenient method for producing diamond semiconductors [19,23]. N-doped n-type diamond semiconductors are often fabricated by adding nitrogen gas into the reaction gas of methane and hydrogen [11,24,25]. However, allowing for the CVD diamond deposition, the N_2_/CH_4_ ratio is limited to a low magnitude (less than 5%) to guarantee that the chemical reactions of diamond growth can take place in the CVD chemical chamber [5]. Hence, depositing the diamond film with a high-concentration N element seems difficult for the CVD technique [26,27]. A previous study reported that a homogeneous N-doped SiO_2_ film was produced on the surface of silicon nitride fibers in dry air at 800–1300 °C [28], which suggested that high temperatures can decompose the surface of silicon nitride to form a new N component for growing N-doped films.

In this work, we demonstrated that super-high-concentration N doping of diamond coatings can be achieved by the simple thermal decomposition of substrate combined with the CVD technique. Generally, N_2_ gas is selected as the N resource to dope into CVD diamond, but problems such as the chemical inertia, low concentration, and low adsorption probabilitycause low-concentration N doping of CVD diamond coatings. Here, the hot filament CVD method was used to provide a high temperature that served as a thermal motivation for obtaining the N and Si resources by pyrolyzing the silicon nitride substrate. In this way, for the N resource, many advantages, including good chemical activity and high adsorption probability due to surrounding the substrate’s surface, are evident for the fabrication of high-concentration N-doped CVD diamond coatings. Furthermore, to enhance the adsorption capacity of the substrate with the N resource, the GO particles were dispersed on the substrate surface to act as the adsorbent. The N-doped CVD diamond coating showed a maximum nitrogen concentration of 47.47%, which is the highest value among the N-doped diamond coatings produced by the CVD method.

## 2. Experimental

Flat Si_3_N_4_ ceramic with square geometry (10 × 10 × 3 mm^3^) produced by the reaction-sintered processing route was selected for examination to deposit the CVD diamond film. Before deposition, the substrate was polished with sandpaper combined with a diamond powder slurry (50 μm) to enhance the nucleation of diamond growth. Then, the substrate was washed with deionized water and high-purity acetone in an ultrasonic container for 10 and 20 min to remove natural impurities on the surface, respectively.

The diamond film was fabricated by a conventional hot filament CVD technology, and the reactor gas was an acetone–H_2_ mixture gas. Four tantalum filaments were selected as hot filaments and straightened by the springs to maintain a constant distance of 10 mm between the hot filaments and the substrate. The temperature of hot filaments during deposition was about 2000 °C, and thus the temperature of the substrate surface was maintained at 800–900 °C. The flow rates of H_2_ and acetone were 240 and 90 sccm, respectively. The deposition process was divided into the nucleation and growth stages. The reaction pressure was set at 2 and 5 kPa in the nucleation and growth stages, respectively. The growth time was set to 0.5, 1, 2, 3, and 4 h to carefully observe the diamond growth.

The surface and cross-sectional morphologies of diamond films with different growth times were characterized by FESEM (Hitachi, Ltd., Tokyo, Japan). The distribution and content of elements in diamond films were tested by EDS. Finally, the quality, internal stresses, and internal defects of each group of films were examined by Raman spectroscopy (Horiba Jobin Yvon Ltd., Paris, France) using a Ne+ laser with an excitation wavelength of 532 nm. X-ray photoelectron spectroscopy (XPS) (Thermo Fisher, Ltd., Waltham, MA, USA) was also employed to evaluate the surface chemical bonding and structure.

## 3. Results and Discussion

### 3.1. Characterization of Diamond Films with Different Growth Times

**Figure 1:**
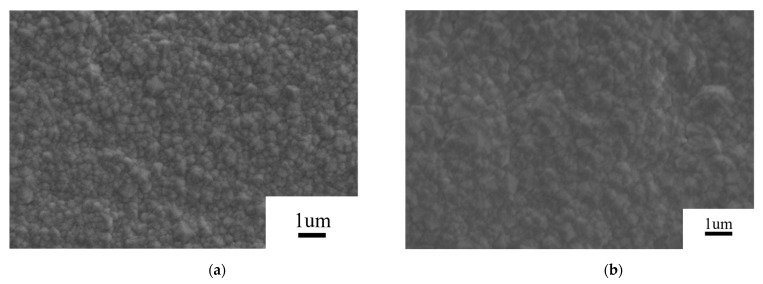

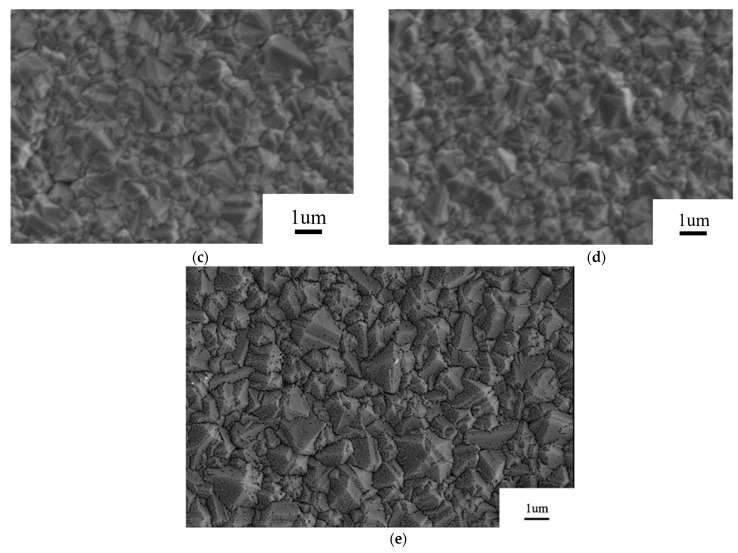
Surface morphologies of diamond films with the growth times of (**a**) 0.5, (**b**) 1, (**c**) 2, (**d**) 3, and (**e**) 4 h.

SEM was conducted to observe the surface morphologies of all the as-deposition diamond films, as shown in Figure 1. For the growth time of 0.5 h, a layer of small diamond particles bonded and covered the whole Si_3_N_4_ surface, which belonged to a heterogenous growth. Thereafter, the diamond grains continued to grow on the as-deposited layer of diamond film, which can be classified as homogeneous growth [29]. The experimental results showed that after 1 h, the relatively larger diamond grains generated on the substrate, which might result from the change in the growth pattern of diamond grains from heterogenous growth to homogenous growth. Obviously, the homogenous growth was more favorable for the diamond fabrication than the heterogenous growth. Interestingly, the size of diamond grains gradually increased with the increase in growth time, as shown in Figure 1c,d. The diamond grains reached about 2 μm after 4 h. 

**Figure 2:**
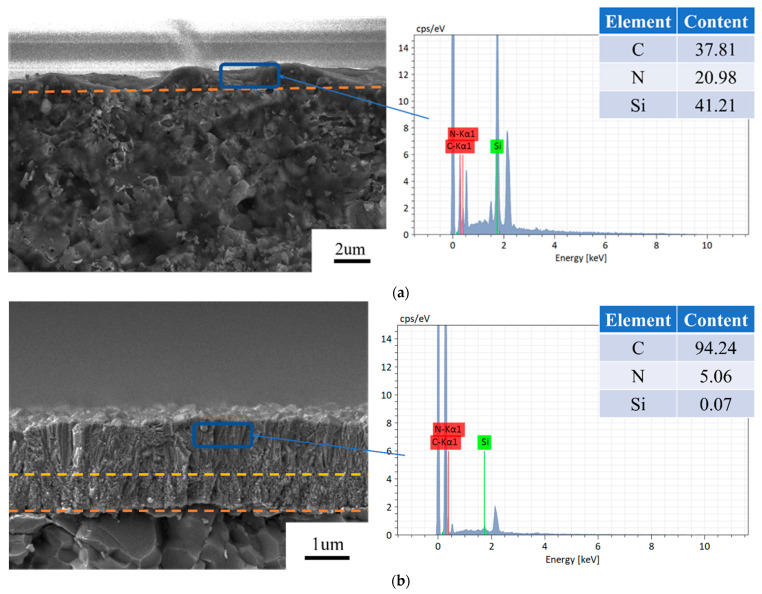

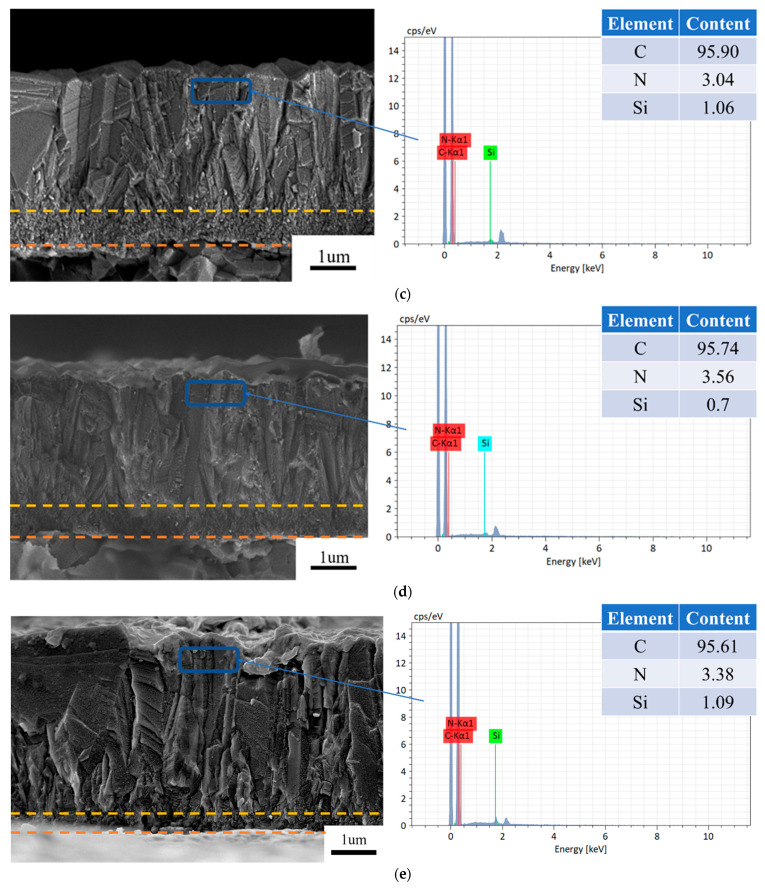
Cross-sectional conditions and elemental distributions of normal MCD films at different growth times: (**a**) 0.5 h (**b**) 1 h (**c**) 2 h (**d**) 3 h (**e**) 4 h.

The cross-sectional surface morphologies of five diamond films with the growth times of 0.5, 1, 2, 3, and 4 h are shown in Figure 2. For the growth time of 0.5 h, the thickness of the diamond film was measured to about 0.63 µm, as shown in Figure 2a. For 1 h, a transition layer can be observed in the cross-sectional profile of the diamond film, which can be divided into two different types of diamond films. The diamond film near the Si_3_N_4_ substrate (S-D film) presented a very small grain size, which was in agreement with the surface morphology. The diamond film with large grains (L-D film) on the upper part of the transition layer exhibited a considerably different morphology as compared with the S-D film, as shown in Figure 2b. The thicknesses of the S-D film and L-D film were about 0.68 and 0.98 µm, respectively. Therefore, it can be inferred that when the thickness of S-D film passes through a threshold, the growth rate of the diamond film remarkably increases. For the growth time of 2 h, the L-D film presented the largest diamond grains, and the thicknesses of the S-D and L-D films were about 0.72 and 2.61 µm, respectively, as shown in Figure 2c. For the growth time of 3 h, the thicknesses of the S-D and L-D films were measured as 0.74 and 3.08 µm, respectively, as shown in Figure 2d. For the growth time of 4 h, the grain size and thickness reached the largest magnitudes, and the corresponding thicknesses were 0.77 and 3.61 µm, respectively. 

It was unexpected that the thickness of S-D film almost maintained a relatively constant magnitude (0.68–0.77 µm). Meanwhile, the growth rate of L-D film appeared to be associated with the growth time. The highest thickness increase of the L-D film reached 1.63 µm per hour during the growth period from 1 to 2 h; thereafter, the growth rate of the diamond film slowed down (such as 3 and 4 h). 

To further obtain the material properties of as-deposited diamond film, EDS was used to investigate the elemental distribution and content of the as-deposited diamond films, as shown in Figure 2. For the growth time of 0.5 h, the N and Si concentrations reached 41.21 and 20.98 at%, respectively, while the C concentration only reached 37.81 at%. Thus, the high-N-concentration doping of diamond film can be achieved, which resulted as completely different from the conventional deposition method by adding N_2_ gas [30]. Since the reactor gas only included carbonous resources and hydrogen, the Si and N atoms in the diamond film certainly derived from the Si_3_N_4_ substrate. According to the CVD chemical reactor condition, high temperatures might lead to the decomposition of Si_3_N_4_ and thus generate the Si and N functional groups that are aggregated near the region of the substrate’s surface [28]. During deposition, these functional groups mixed the carbonous resources, and hydrogen easily doped into the diamond film. This may be the reason for the high Si and N concentrations of the S-D film. Meanwhile, as the thickness of the film increased, the substrate was fully covered by the S-D film, resulting in the inhibited decomposition of Si and N functional groups from the Si_3_N_4_ substrate. Thereafter, a rapid decrease in the Si and N concentrations in L-D film was observed for the diamond film the growth times of 1, 2, 3 and 4 h, as shown in Figure 2b–e. The C concentrations of these diamond films reached up to about 95 at%, while the Si and N atoms were also found in the L-D film due to the residual Si and N functional groups in the reactor gas.

**Figure 3:**
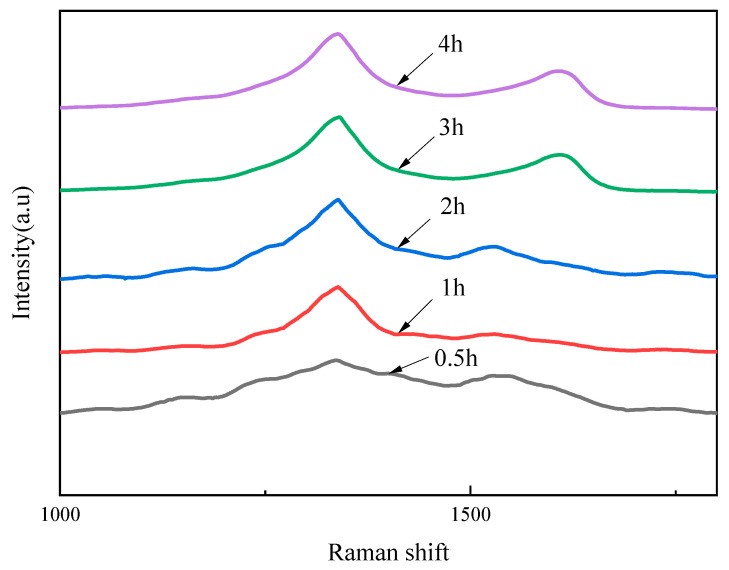
Raman spectra of as-deposited diamond films.

Raman spectroscopy was conducted to further investigate the diamond phase and purity of the as-deposited diamond films, as shown in Figure 3. For all the diamond films, a remarkable Raman peak was observed near the natural diamond Raman peak of 1332 cm^−1^, which indicated that a diamond phase was generated on the Si_3_N_4_ substrate [31]. However, these peaks presented a deviation from 1332 cm^−1^ with a different level, which was attributed to the change of crystalline parameters due to the internal stress. The diamond peaks of as-deposited diamond films with the growth times of 0.5, 1, 2, 3, and 4 hwere located at 1335.67, 1337.8, 1337.8, 1330.25, and 1336.76 cm^−1^, respectively. The internal stresses of the corresponding diamond film were calculated to be −2.08, −3.289, −3.289, 0.992, and −2.6989 GPa by the equation *σ*(GPa) = *α*Δ*v* = −0.567 (*v* − *v*_0_), where Δ*v* is the difference between the measured peak of as-deposited diamond and the characteristic peak of natural diamond [32]. On the other hand, the other Raman peaks located at 1540–1600 cm^−1^, which were formulated as the G band [33], were associated with the growth of graphite-like sp^2^ bonded components at the grain boundary of the diamond film.

**Figure 4:**
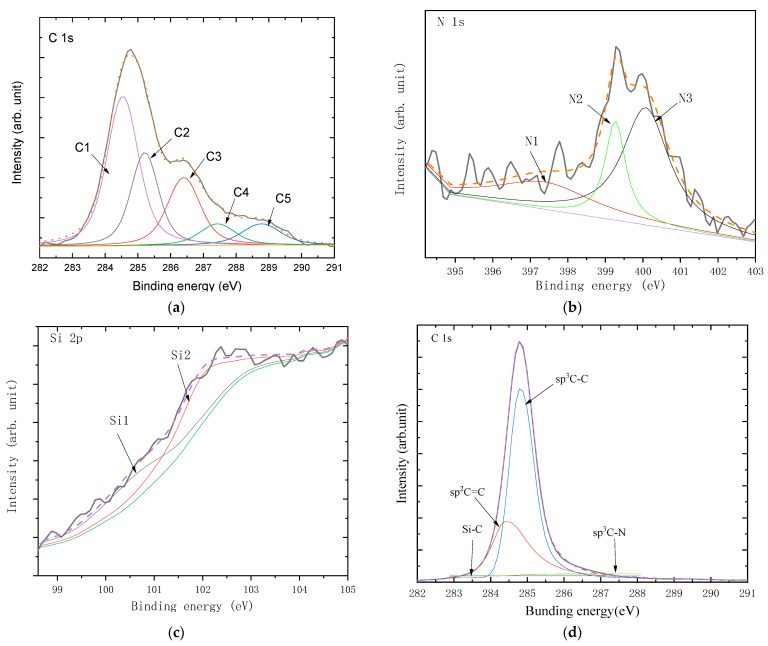
(**a**) C1s, (**b**) N1s, and (**c**) Si 2p XPS spectra of diamond films with the growth time of 0.5 h; (**d**) C1s XPS spectra of diamond films with a growth time of 4 h.

XPS was used for detecting the transformation of the surface chemical state and determining the possible formation of new bonds. Figure 4 shows the C 1s, N 1s, and Si 2p spectra of the diamond films with growth times of 0.5 and 4 h, where the deconvolution of the high-resolution spectra was also given and the background subtraction was set as Shirley-type. The spectrum of diamond film with a growth time of 0.5 h revealed that C, N, and Si elements accounted for 84.46 at%, 5.23 at%, and 5.31 at%, respectively. The C 1s spectrum was decomposed into five components, viz., C1, C2, C3, C4, and C5, located at 284.5, 285.2, 286.4, 287.4, and 288.7 eV, respectively, which were associated with the sp^2^ C=C [34], sp^3^ C-C [35,36], sp^2^ C-N/C=N, sp^3^ C-N, and C-OOH bonds [37]. The larger peak area ratio of the C1 component (46.7%), as compared with that of the C2 component (18.29%), indicated that the diamond film contained many nondiamond bonds, which might be attributable to the chemical reaction of the N atom that existed in the CVD chamber because the C2 and C3 bonds with a 29.0% peak area ratio formed between the C atom and N atom and in turn produced several sp^2^ C bonds, rather than sp^3^ C bonds [38,39]. Here, the peak located at ~283.2 eV (associated with Si-C bond) was not found in the XPS spectrum, suggesting that the amount of Si-C bond was very small in the diamond film [40]. For the N 1s spectrum, three peaks of N1, N2, and N3 components were decomposed at 397.4, 399.3, and 400.1 eV, which were assigned to Si-N, C-N, and C=N bonds, respectively. These data suggested that the N atom interacted with the Si atom, which might derive from two possible approaches: (1) the original Si-N chemical group being thermally decomposed from the Si_3_N_4_ substrate, and (2) the Si-N chemical bond being regenerated again. On the other hand, the C-N and C=N bonds were also found in the N 1s spectrum, which were consistent with those in the C 1s spectrum. For the Si 2p spectrum, two peaks of Si1 and Si2 components located at 100.9 and 102.0 eV were associated with the Si-C and Si-N bonds, respectively. This further demonstrated that a Si-N bond existed in the diamond film [41]. Here, The Si-C component decomposed from the Si 2p spectrum was not observed in the N 1s spectrum, which was likely caused by the small amount of Si-C bond in the diamond film.

For the diamond film with a growth time of 4 h, the XPS spectra of the diamond films showed 98.33 at%, 0.36 at%, and 1.32 at% of C, N, and Si elements, respectively. Its C 1s spectrum was decomposed into four components, viz., Si-C, sp^2^ C=C, sp^3^ C-C, and sp^3^ C-N, located at 283.3, 284.4, 285.0, and 287.3, respectively. The larger peak area ratio of the sp^3^ C-C component (68.2 at%), as compared with that of the sp^2^ C=C component (29.4 at%), indicated that the diamond film contains a small amount of sp^2^ nondiamond bonds, which is similar to the results of the Raman spectrum in Figure 3.

### 3.2. Characterization of Diamond Films with GO Particles

**Figure 5:**
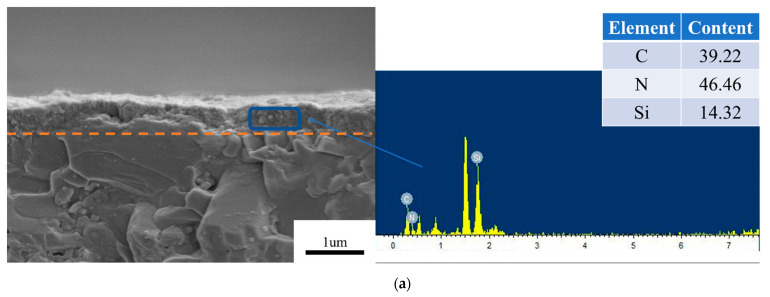

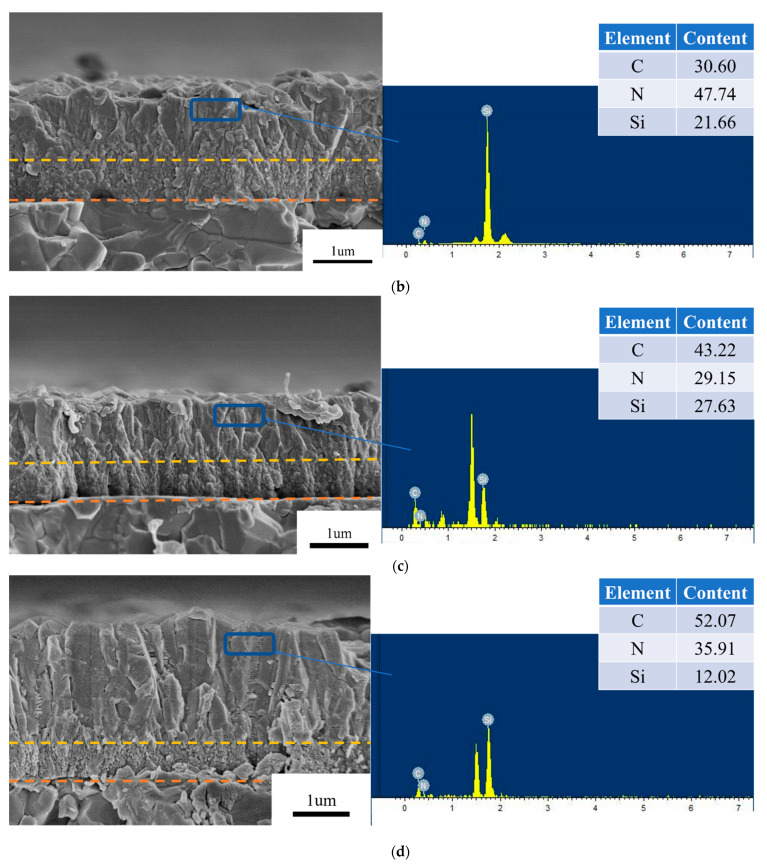

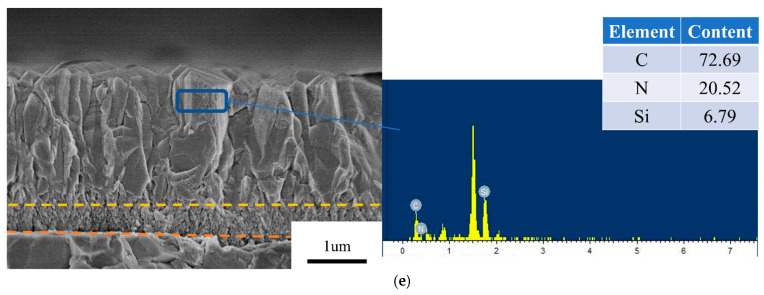
Cross-sectional profiles and elemental distributions of GO-MCD films with the growth times of (**a**) 0.5 h, (**b**) 1 h, (**c**) 2 h, (**d**) 3 h, and (**e**) 4 h.

As mentioned above, the growth time played a vital role in the diamond phase and surface morphology of the diamond films. During the CVD chemical reactions, the reactor gas was always pumped into the chemical chamber to compensate for the consumption of reactor gas, and the exhaust gas, including the decomposed N and Si components, was also continuously emitted from the chemical chamber by the pump. Hence, avoiding the low-concentration gaseous N components near the substrate was required for depositing the diamond doped with the high-concentration N element. In this way, the high growth rate of diamond might be a preferable option to achieve high-concentration N doping of diamond, since the decrease in growth time was indicative of the increase in concentration of the N component near the substrate. In the previous literature [26], adding GO particles on the Si_3_N_4_ surface effectively increased the growth rate of diamond film. The deposition process of GO-diamond film was the same as in the literature [42]. The GO particles were added to the substrate surface after pretreatment of the substrate by a diamond powder slurry. The Raman spectra demonstrated that a small internal stress can be observed for the GO-diamond film. Here, the cross-sectional morphologies were characterized by an analysis of EDS to study the evolutional tendency of the distribution of Si and N atoms along the thickness direction, as shown in Figure 5. The same growth time of 0.5, 1, 2, 3, and 4 h was set to produce the GO-diamond films for comparison. For the growth time of 0.5 h, the S-D film with small diamond grains still existed, and its thickness reached 0.75 μm. However, as compared to the pure diamond film, the converse tendency of the concentration of Si and N atoms in the diamond film was found for the GO-diamond film. The weight content of Si and N atoms reached 14.31 and 46.46 at%, respectively. Here, a rapid increase in the concentration of the N atom in the diamond film might result from the stronger adsorption capacity of GO particles for the N atom relative to the Si atom during the CVD diamond growth [43,44]. For the growth time of 1 h, the distinct grain boundary between S-D film and L-D film still presented in the GO-diamond film, which was similar to that of the pure diamond film. The diamond grains grew up to a larger micro-scale size and were covered on the S-D film. The thicknesses of S-D and L-D films were measured to be 1.04 and 2.12 um, respectively. On the other hand, the weight contents of Si and N atoms were up to 21.66 and 47.47 at%, respectively, which were definitely different from the pure diamond film where the corresponding content of Si and N atoms rapidly decreased to 0.07 and 5.06 at%. To the best of our knowledge, the percentage of 47.47 at% set a new remark for the high-concentration N doping of diamond film. As the growth time increased, the concentration of Si and N atoms in the diamond film generally decreased because of the reduction of the amount of Si and N atoms in the reactor gas. For the growth time of 4 h, the weight contents of the N atom (20.52 at%) and Si atom (6.79 at%) still existed for the diamond film, which were considerably larger than those of the pure diamond film. Hence, enhancing the growth rate by adding Go particles on the Si_3_N_4_ substrate might be classified as a convenient and simplified method to achieve high-concentration N and Si doping of diamond film.

**Figure 6:**
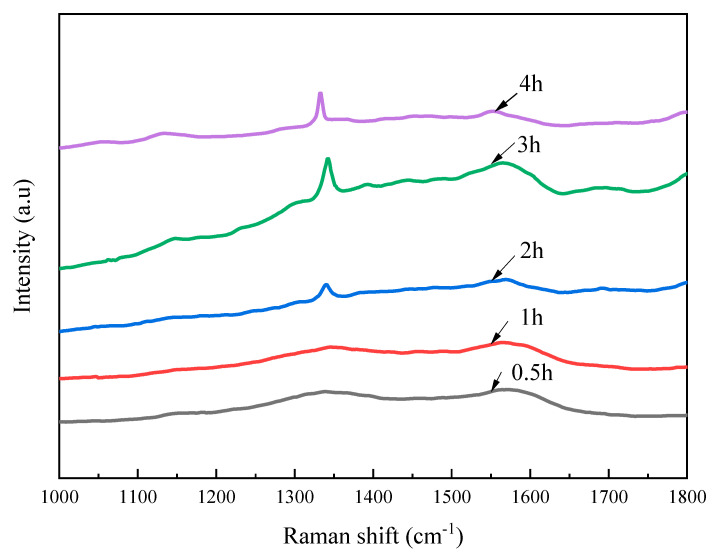
Raman spectra of the GO-diamond films.

Figure 6 shows the Raman spectra of as-deposited GO-diamond films. The diamond peaks of GO-diamond films with the growth times of 0.5, 1, 2, 3, and 4 h were located at 1331.83, 1342.98, 1338.2, 1346.16, and 1338.2 cm^−1^, respectively. The number of diamond phases of the GO-diamond film increased with increasing growth time, which might be a result of the decrease in the content of the N and Si components in reactor gas. For the growth time of 4 h, the low peak of the G band indicated that non-diamond phases were very low in concentration in the diamond film. As compared with the pure diamond film, the secondary peak in the G band (2260 cm^−1^) was not observed in the GO-diamond film.

**Figure 7:**
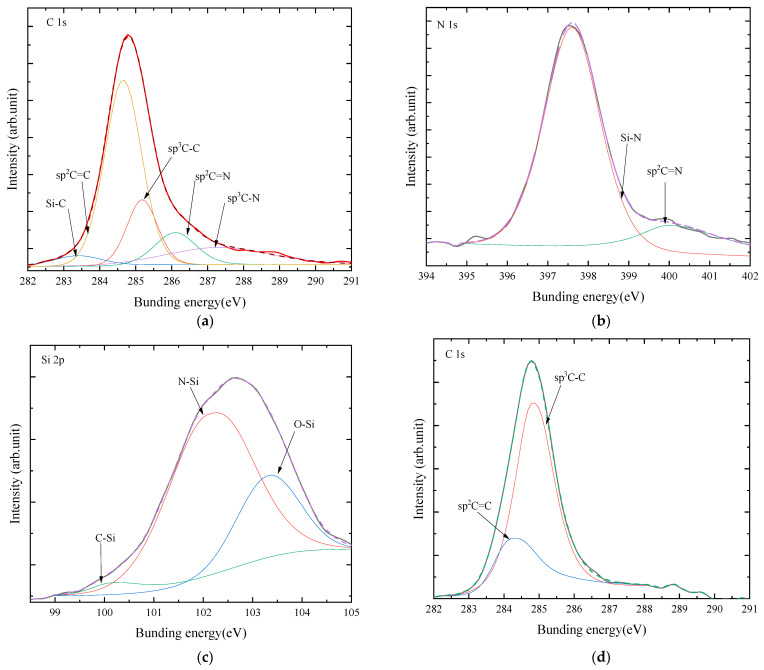
(**a**) C1s, (**b**) N1s, and (**c**) Si 2p XPS spectra of the GO-diamond film with a growth time of 0.5 h; (**d**) the C1s XPS spectrum of GO-diamond film with a growth time of 4 h.

The XPS spectra of GO-diamond films with growth times of 0.5 and 4 h are shown in Figure 7. The spectrum of the GO-diamond film with a growth time of 0.5 h revealed that the C, N, and Si elements accounted for 65.12 at%, 12.79 at%, and 22.09 at%. The C 1s spectrum was decomposed into five components, viz., Si-C, sp^2^ C=C, sp^3^ C-C, sp^2^ C=N, and sp^3^ C-N, located at 283.3, 284.4, 285.3, 286.1, and 287.3 eV, respectively. The peak area ratio of the sp^2^ C=C component (45.3%) was much larger than that of the sp^3^ C-C component (15.4%). For the N 1s spectrum, two peaks of Si-N and sp^2^ C=N components were decomposed at 397.8 and 400.1 eV, respectively. The peak area ratio of the Si-N component reached 89.2%. For the Si 2p spectrum, three peaks of N-Si, C-Si, and O-Si components were located at 100.1, 102.1, and 103.3 eV, respectively. This indicates that most of the Si atoms in the diamond film mainly interact with the N atoms to form Si-N bonds.

For the GO-diamond film with a growth time of 4 h, the XPS spectrum suggested that the C, N, and Si elements accounted for 96.00 at%, 3.99 at%, and 0.01 at%, respectively. Its C 1s spectrum was decomposed into two components of sp^2^ C=C and sp^3^ C-C, located at 284.4 and 284.8, respectively. The peak area ratio of the sp^3^ C-C component increased to 72.3%.

## 4. Conclusions

A novel CVD method combined with the thermal decomposition of a substrate was proposed in this work to increase the concentration of the N element in diamond film. As compared to the normal technique of the N_2_ addition into reactor gas, the N and Si components with strong chemical activations that decomposed from the substrate at high temperatures more easily reacted with the carbonous resource under hydrogen conditions to achieve high-concentration N and Si doping of diamond film.

The growth time considerably affected the surface morphology of pure diamond film. Firstly, the diamond grains were too small to match with the substrate. As the growth time increased, the diamond grain became larger and larger. However, a remarkable boundary was found at the thickness of 0.72–0.77 µm of diamond film, which might be a result of a distinct difference in the diamond grains. The experimental observation demonstrated that the diamond coating with a growth time of 0.5 h contained N and Si elements with high concentrations of 20.78 and 41.21%, respectively. However, they decreased to 3.38 and 1.09% for the growth time of 4 h, which also led to the increase of the diamond phases with the increasing of the growth time. For the chemical bonds in the diamond film, the N atom interacted with the C and Si atoms to form the C-N, C=N, and Si-N bonds, while there was no evidence that the Si atom bonded with the C atom since the number of Si-C bonds was very low.

GO particles were employed to further increase the concentration of N and Si elements in diamond film by improving its growth rate; the GO-diamond film exhibited a higher concentration of N than the pure diamond film. For the growth time of 0.5 h, the contents of N and Si elements reached 47.47 and 21.66%, respectively which set a new record for high-concentration N doping of diamond film. Therefore, the free radical motivated by the thermal decomposition of the substrate can be considered as an alternative and promising technique to deposit super high-concentration N and Si elements onto diamond films, which provides bright prospects for the application of diamond devices in electric fields.

## Data Availability

Not applicable.
